# Association Between TP53 Mutations and Platinum Resistance in a Cohort of High-Grade Serous Ovarian Cancer Patients: Novel Implications for Personalized Therapeutics

**DOI:** 10.3390/ijms26052232

**Published:** 2025-03-01

**Authors:** Clelia Madeddu, Eleonora Lai, Manuela Neri, Elisabetta Sanna, Giulia Gramignano, Sonia Nemolato, Mario Scartozzi, Sabrina Giglio, Antonio Macciò

**Affiliations:** 1 Medical Oncology Unit, Department of Medical Sciences and Public Health, University Hospital Cagliari, University of Cagliari, 09100 Cagliari, Italy; ele.lai87@gmail.com (E.L.); marioscartozzi@gmail.com (M.S.); 2 Gynecologic Oncology Unit, ARNAS G. Brotzu, Department of Surgical Sciences, University of Cagliari, 09100 Cagliari, Italy; manu.neri11@hotmail.it (M.N.); dr.elisabettasanna@gmail.com (E.S.); 3 Medical Oncology Unit, San Gavino Hospital, 09037 San Gavino, Italy; giuli.gramignano@gmail.com; 4 Department of Pathology, ARNAS G. Brotzu, 09100 Cagliari, Italy; sonia.nemolato@aob.it; 5 Medical Genetics Unit, Department of Medical Sciences and Public Health, R. Binaghi Hospital, University of Cagliari, ASL 8, 09100 Cagliari, Italy; sabrina.giglio@unica.it

**Keywords:** platinum sensitivity, BRCA mutations, p53 mutation, ovarian cancer, PARP inhibitors, cisplatin

## Abstract

The integrity of p53 machinery is crucial for platinum activity, while p53 mutation is frequent in high-grade serous ovarian cancer (HGS-OC). This study aimed to evaluate the link between p53 mutations, platinum sensitivity (PS), and the platinum-free interval (PFI) in patients with HGS-OC. We prospectively analyzed 159 consecutive women with ovarian cancer who underwent surgery. The somatic mutational status of BRCA, HRD, and TP53 (according to structural, hotspot, and functional classification) was evaluated. Among enrolled patients, 82.4% of cases were TP53-mutated (MT), and 27.8% were BRCA-MT. The distribution of TP53 mutation categories did not differ significantly between the BRCA-MT and wild-type (WT) cases. In the entire population, the proportion of PS patients was significantly lower in TP53-MT compared to TP53-WT (*p* = 0.0208), in nonsense/frameshift/splicing compared to missense (*p* = 0.0319), and in loss-of-function (LOF) compared to GOF (*p* = 0.0048) MT cases. For the BRCA-MT patients, structural and functional TP53 mutations were not significantly different between the PS and PR patients. Conversely, for the BRCA WT patients, the distribution of structural and functional TP53 mutations significantly differed between PS and PR patients. In a multivariate regression analysis, LOF mutations were found to be independent negative predictors of PS (HR: 0.1717; 95% CI: 0.0661–0.4461; *p*-value: 0.0003). Kaplan–Meier curves showed a significantly lower PFI in cases with LOF mutations in the overall population (log-rank *p* = 0.0020) and in BRCA-WT patients (log-rank *p* = 0.0140). Via multivariate COX testing, it was found that LOF mutations were independently associated with a decreased PFI (*p* = 0.0036). In conclusion, our data show that HGS-OC harboring p53 LOF mutations is the poorest prognostic subgroup regarding PS and the PFI. Further studies are needed to confirm our findings.

## 1. Introduction

The standard treatment for high-grade serous ovarian cancer (HGSOC) involves cytoreductive surgery followed by platinum-based chemotherapy [1]. Although HGSOC is typically platinum-sensitive [2], most patients develop resistance to chemotherapy and, unfortunately, pass away from their disease within five years of diagnosis [3]. Platinum sensitivity influences prognosis, while resistance to primary chemotherapy results in treatment failure and unfavorable outcomes [4]. Sensitivity is crucial for PARP inhibitor (PARPi) effectiveness in advanced HGSOC. These agents revolutionized HGSOC therapy, significantly improving prognosis. In the SOLO-1 trial, Olaparib reduced disease progression or mortality risk by 70% versus a placebo [5]. PARP inhibitors are now recommended as maintenance therapy after platinum-based treatment for ovarian cancer, both in first-line and recurrent settings.

Both somatic and germline BRCA mutations are crucial in predicting sensitivity to platinum-based chemotherapy in HGSOC [6]. This is because the lack of a DNA repair mechanism enhances the effectiveness of platinum as a DNA alkylating agent. These mutations have also become predictive markers for the efficacy of PARPi due to their capability to forecast the response to platinum salts [7,8]. Their enhanced effectiveness in BRCA-mutated ovarian cancer (OC) is notably demonstrated by extended progression-free survival (PFS) and overall survival (OS) when compared to BRCA wild-type OC across various treatment settings, including both initial and recurrent disease [9]. Moreover, PARP inhibitors (PARPi) have a response rate exceeding 40% in BRCA 1-2 mutated OC. The BRCAness phenotype is associated with impaired homologous recombination (HR), due to factors like epigenetic hypermethylation of BRCA1, somatic BRCA mutations, EMSY amplification, and deficits in other HR pathway genes [10]. This highlights the role of BRCA and related genes in HR, influencing outcomes and treatment options for HGSOC. Notably, a defective HR pathway in BRCAness patients, even without BRCA mutations, shows increased sensitivity to DNA-damaging agents such as cisplatin and PARPi.

The cytotoxic effects of CDDP and PARP are primarily mediated through various mechanisms, including DNA damage, but also extend beyond it, leading to cell death via p53-mediated apoptosis [11]. Maintaining the integrity of p53 machinery is essential for platinum-related cytotoxicity [12]. In contrast, p53 mutations linked to various protein expressions commonly occur in ovarian cancer, particularly in HGSOC and BRCA-mutated tumors [10].

TP53, on chromosome 17P13, known as the “guardian of the genome” or “cellular gatekeeper”, is crucial for tumor suppression by regulating downstream genes. This regulation triggers cellular responses like cell cycle arrest and apoptosis in reaction to stressors such as DNA damage, hypoxia, and nutrient deprivation [13]. TP53 mutations occur in about 90% of HGSOC cases, correlating with metastatic progression and chemotherapy resistance, as shown by next-generation sequencing (NGS) [14]. Approximately 95% of somatic p53 mutations are found in the core DNA-binding domain, with 80% being missense mutations leading to mutant p53 (mutp53) overexpression, detectable via immunohistochemistry. In contrast, frameshift, nonsense, and splice-site mutations (p53-null mutations) result in the absence of protein. These mutations can lead to loss of wild-type (WT) P53 function (LOF) and gain of function (GOF) of mutated P53, contributing to carcinogenesis, multidrug resistance, poor prognosis, and metastasis [15].

Few studies have examined the role of p53 in mediating platinum sensitivity and the response to PARP in patients with HGSOC. Investigations into platinum resistance mechanisms in rare low-grade ovarian cancers increasingly focus on mutational profiles, revealing a link to p53 mutations. This expands molecular profile analyses from BRCA mutations to include p53 mutations in HGSOCs.

Genomic analyses provide crucial insights, particularly on platinum sensitivity predicting PARP inhibitor response in optimally debulked HGSOC cases without residual disease. In such cases, determining platinum sensitivity based on standard criteria [16] at the conclusion of adjuvant chemotherapy within a six-month interval is challenging. The absence of disease may significantly depend on the extent of surgery and the effectiveness of adjuvant therapies, including cisplatin, paclitaxel [17], and occasionally bevacizumab. Serum markers or instrumental evaluations are not feasible due to the short observation period following surgery chemotherapy. Therefore, potential predictive markers for lack of efficacy and platinum resistance—such as the p53 mutation, when considering the type of mutation and the related functional alteration of the protein—along with the analysis of the BRCA and HRD profiles, could improve patient selection for initiating maintenance therapy and ultimately lead to a personalized decision regarding the most effective maintenance treatment.

Few studies have examined the correlation between p53 mutations and platinum responses in HGSOCs, resulting in mixed findings [15,18,19,20]. Research using preclinical mouse models suggests that the loss of p53 may lead to resistance to PARP inhibitors in BRCA1-mutant cells [21].

Starting from the above background, we investigated the correlation between p53 mutations and platinum response. We aimed to determine if we could pinpoint, based on the specific p53 mutation and the related changes of function/activity, which patients affected by HCSOC are likely to be platinum-sensitive and, therefore, suitable for PARP inhibitors and select the most effective maintenance therapy promptly and efficiently.

## 2. Results

Among the entire cohort, only patients with HGS-OC were prospectively included in the analysis for a total of 159 patients (Table 1). As regards ethnicity, all patients were Caucasian. Most (149, 93.6%) presented with stage III-IV disease (Table 2). Regarding surgery, 74% underwent initial cytoreductive surgery with radical intent (optimal cytoreductive surgery). The remaining patients (26%) were not radically/optimally resectable and underwent diagnostic laparoscopy with minimal surgery (isolated omentectomy, adnexectomy, or peritoneal biopsy) to obtain a histological diagnosis. After surgery, all primary serous ovarian carcinoma patients received cisplatin-based chemotherapy according to standard guidelines. TP53 mutations were detected in 131 patients (82.4%) within the entire cohort; 6 patients were not assessed for p53 mutations, and 22 patients exhibited TP53 wild-type status (Table 1). Noteworthy, all histologies of these 22 cases were high-grade serous ovarian cancer. Somatic BRCA mutations were identified in 44 (27.7%) patients. The stage and optimal cytoreduction distribution were superimposable between the BRCA-mutated and BRCA wild-type patients. The frequency of TP53 mutation was not significantly different between the BRCA-mutated and BRCA wild-type patients (*p* = 0.4520). Mutations of other HRD genes (excluding BRCA mutations) were identified in 16 cases (10.1%) (Table 2) without significant difference in distribution among the BRCA wild-type (10.4%) and BRCA-mutated (9.1%) patients (*p* = 0.7733) (Table 1). Among the BRCA-mutated patients, a higher percentage of platinum-sensitive cases was observed (72.7% versus 52.2%, *p* = 0.0445) (Table 1). Employing the structural classification scheme, most cancers harbored a missense TP53 mutation (94/131; 71.8%). According to the functional classification scheme, GOF TP53 mutations were identified in 70 cases (56.5%), while LOF TP53 mutations were present in 43 patients (34.7%). Approximately 8.9% of the mutations in our cohort remained unclassified according to the International Agency for Research on Cancer classification scheme. Supplementary Figure S1 reports a lollipop plot showing the location and frequency of p53 mutations in our cohort (Figure S1). No significant disparity was observed in the distribution of specific TP53 mutations within each classification scheme (i.e., missense vs. nonsense/frameshift/splice and GOF vs. LOF) between cases with BRCA mutations and those without (Table 2). A higher incidence of hotspot mutation was observed in BRCA-mutated tumors (*p* = 0.0461) (Table 2).

### 2.1. Assessment of Platinum Sensitivity and PFI According to BRCA and p53 Mutational Status

Overall, 92 (57.9%) patients were classified as platinum-sensitive, while 67 (42.1%) patients were platinum-resistant/refractory (Table 1). The percentage of platinum-sensitive patients was higher among those with BRCA mutated tumors compared to BRCA WT cases (72.7% versus 52.2%, *p* = 0.0445). Considering the entire population, including both BRCA-mutated and BRCA WT tumors, we found that the percentage of platinum-resistant patients was significantly higher in those with p53-mutated tumors in comparison p53 wild-type tumors (*p* = 0.0208). Moreover, the percentage of platinum-sensitive patients was significantly lower in those with nonsense/frameshift/splicing mutations compared with those with missense mutations (*p* = 0.0124) and in those with LOF mutations compared with those with GOF mutations (*p* = 0.0032) (Table 3).

In the context of the BRCA-mutated patients, examining the association of BRCA1/2 mutations and p53 mutations with response to platinum and platinum-free intervals, we observed that the individuals without p53 mutations (p53 WT) all exhibited platinum sensitivity. In contrast, among those with p53 mutations, 28 patients were platinum-sensitive, and 10 patients were platinum-resistant/refractory (*p* = 0.2455). Regarding the distribution of platinum sensitivity based on the structural classification of p53 mutations, no significant differences were observed between missense and nonsense/frameshift/splice mutations (*p* = 0.2586) (Table 3). Moreover, we noted that GOF mutations were linked to platinum sensitivity in 15 patients and platinum resistance in 4 patients. Conversely, LOF mutations were associated with platinum sensitivity in six patients and platinum resistance in four patients. The disparity in the distribution of platinum sensitivity across functional categories of p53 mutations was not statistically significant (*p* = 0.2863).

In the BRCA wild-type cohort of HGSOC patients, we observed that patients without p53 mutations were mostly platinum-sensitive (14 out of 18, 63.6%), while among those with p53 mutations, 42 (32.8%) were platinum-sensitive and 51 (37.4%) were platinum-resistant/refractory (*p* = 0.0117). Among those with p53 mutations, a significant difference was detected in platinum sensitivity across the various categories of structural classification of p53 mutations, with a higher percentage of platinum-resistant/refractory cases among cases with nonsense/frameshift/splice mutations (*p* = 0.0177). Furthermore, GOF mutations were linked to platinum sensitivity in 30 patients and platinum resistance in 21 patients. In comparison, LOF mutations were associated with platinum sensitivity in 10 patients and platinum resistance in 23 patients (*p* = 0.0111). Of particular note, in five cases, we identified that LOF mutations associated with null expression of HIC p53 were observed in those patients who exhibited refractoriness to platinum-based chemotherapy.

We found that the PFI was significantly lower in patients with the TP53 mutation, in the entire population, and both BRCA WT and BRCA MT groups (Table 3). Moreover, the presence of TP53, LOF mutation was associated with a significantly lower PFI in comparison to patients with TP53 GOF mutation in the entire population and the BRCA WT subgroup (Table 2).

Via the logistic regression analysis, we found a significant negative association between platinum sensitivity and p53-mutated status, nonsense/frameshift/splicing structural status, LOF mutation, and non-hotspot mutations in the overall population and in BRCA wild-type cases, but not in the BRCA-mutated patients (Table 4). Via the multivariate regression analysis, LOF mutational status retained a significant independent negative association with platinum sensitivity (overall population: r coefficient: −1.76191; HR: 0.1717; 95% CI: 0.0661–0.4461; *p*-value: 0.0003; BRCA wild-type cases: r coefficient −1.89712; HR: 0.1500; 95% CI: 0.0469–0.4795; *p* = 0.0014).

### 2.2. Platinum-Free Interval by BRCA and P53 Mutational Status

**Figure 1:**
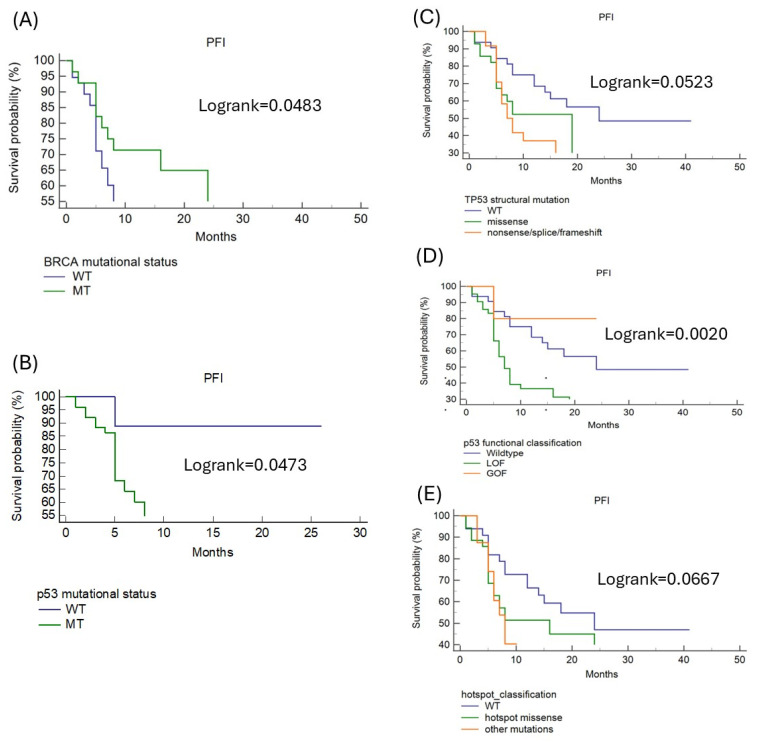
Platinum-free interval comparison according to BRCA and TP53 mutational status in overall population: (**A**) BRCA mutational status, (**B**) TP53 mutational status (WT = wild-type, MT = mutated), (**C**) functional classification (LOF = loss of function, GOF, gain of function), (**D**) structural mutation class, (**E**) hotspot classification.

A germline or somatic BRCA mutation did confer an increase in the PFI (log-rank *p* = 0.0483, Figure 1A). The PFI also significantly differed between cases with (median PFI 7 months) and without (median PFI 12 months) TP53 mutations (log-rank *p* = 0.0473, Figure 1B). Using the structural classification, the median PFI was 24 months in cases with wild-type TP53, 19 months with missense mutations, and 7 months with nonsense/frameshift/splice mutations (log-rank *p* =0.0523, Figure 1C). Using the functional classification, cases with GOF TP53 mutations demonstrated a significantly higher median PFI of 20 months, while LOF TP53 mutations demonstrated a median PFI of 7 months (log-rank *p* = 0.0020, Figure 1D). Cases with hotspot missense TP53 mutations had a median PFI of 16 months compared to a mean PFI of 8 months for cases with non-hotspot mutations (log-rank *p* =0.0667, Figure 1E). To analyze further the potential contribution of TP53 mutations in predicting PFI, cases were stratified by the presence of BRCA mutations. For cases with somatic BRCA mutations, there was no significant difference in PFI curves between cases with or without TP53 mutations (Figure S2A). Vice versa, BRCA wild-type cases showed a significant difference in the PFI based on the presence of any TP53 mutation (log-rank *p* = 0.0244), LOF mutations (log-rank *p* = 0.0140), and nonsense/splice/frameshift mutations (log-rank *p* = 0.0457) (Figure S2B).

In multivariate Cox testing of the associations between TP53 mutations and the PFI, LOF mutations were independently associated with a decreased PFI (*p* = 0.0036) (Table 5).

## 3. Discussion

In the present prospective cohort study, we assessed the role of p53 mutations, classified based on structural and functional alteration, as potential predictive markers of lack of efficacy and platinum resistance in advanced HGSOC patients. The prediction of platinum sensitivity in such patients is crucial to establish the choice of the best first-line chemotherapy and maintenance treatment. In fact, while platinum chemotherapy remains a cornerstone in contemporary treatment, the grim reality persists that the majority of women diagnosed with OC will inevitably develop platinum resistance. Among these patients, approximately 20–30% who initially respond well to treatment will experience recurrence within six months following the completion of primary platinum therapy [22]. However, a subset of patients, constituting around 16% of those with serous histology, exhibit survival exceeding 10 years. Conversely, there are others diagnosed with the same disease stage and treated with similar therapeutic modalities who endure rapid disease progression. Regrettably, current clinical algorithms cannot accurately predict patient survival outcomes at the time of diagnosis, resulting in the administration of uniform treatments [3]. Age at diagnosis, disease stage, grade, histology, residual disease post-surgery, and disease recurrence have all been identified and validated as having prognostic significance [23]. However, with the introduction of novel maintenance treatment strategies, an urgent need arises to elucidate the mechanisms underlying platinum and PARPi resistance in HGSOC to refine patient stratification for therapeutic interventions targeting molecular vulnerabilities, thereby overcoming treatment resistance. Notably, resistance to platinum chemotherapy strongly foretells resistance to PARPi treatment. The phenomenon of cross-resistance between these two distinct therapeutic classes implies an overlap in the biological mechanisms governing susceptibility and resistance [23].

Presently, the sole markers established and validated to predict an enhanced response to platinum agents are linked to mutations in BRCA1/2 and homologous repair deficiency in HGSOC. Decades of research have shown that mutations in BRCA1, BRCA2, and other genes involved in homologous recombination (HR) are significant prognostic indicators for survival and response to platinum-based therapy in ovarian cancer [24,25]. Indeed, deficits in DNA repair pathways attributed to BRCA1/2 and HR system mutations render them vulnerable to DNA-damaging therapies such as platinum [26,27] and PARP inhibitors [6,8,28]. However, beyond the BRCA1/2 mutation and HRD status, no other biomarker enables the upfront and precise identification of patients with platinum-sensitive or platinum-resistant disease. As such, the underlying disease biology does not inform initial treatment plans [3].

Several mechanisms of cytotoxicity for both CDDP and PARP inhibitors involve apoptosis, which depends on the maintenance functioning of p53 [22]. Notably, mutations in p53 are found in more than 80% of EOC, with the highest frequency in HGSOC [17]. Therefore, it is critical to study the role of p53 in predicting sensitivity or resistance to platinum-based chemotherapy and the potential interactions between TP53 and BRCA mutations as determinants of response to therapy and outcomes [29].

In the present study, we found that TP53 mutations were detected in approximately 82% of patients in the entire cohort without a difference in frequency between BRCA-mutated and BRCA wild-type patients. Employing the structural and functional classification scheme, most cancers harbored missense (71.7%) and GOF (56.5%) TP53 mutations without a significant disparity between cases with BRCA mutations and those without.

Some studies have investigated the association between TP53 mutations and platinum responses in ovarian cancer; however, the results have been discordant and derived from heterogenous studies, neither of which accounted for the BRCA mutation status [3,14,18,30,31,32].

Vice versa, our present study analyzed the influence of the presence and type of p53 mutation on responsiveness to platinum by stratifying patients according to BRCA status. We found that p53 mutation was negatively associated with platinum sensitivity in the entire cohort. Among the BRCA-mutated patients, those without p53 mutations (p53 WT) all exhibited platinum sensitivity. Among the BRCA wild-type patients, individuals without p53 mutations also demonstrated a significantly higher platinum sensitivity than those with p53 mutations. The presence of p53 mutations was also associated with a significantly lower PFI in the entire cohort and both BRCA wild-type and BRCA mutant patients.

In a recent large single-institution cohort study of OC patients conducted by Ghezelayagh et al. [33], TP53 mutations were found to be associated with primary platinum sensitivity in high-grade serous carcinoma (HGSC), irrespective of the specific mutation type, after adjusting for covariates, including BRCA mutation status. The study conducted by Ghezalayagh et al. [33] was quite similar to our current research; however, it included both serous and non-serous ovarian carcinomas and aimed to examine the impact of p53 mutations on overall survival rather than on the PFI, i.e., progression-free survival, which is a focus of our study. In this regard, our results, when compared to the findings obtained by Ghezalayagh et al. in the subset of patients with HGSC, are not superimposable: indeed, we identified a significant difference in the PFI based on the presence of any p53 mutation in both BRCA WT and BRCA-mutated patients. Additionally, our Kaplan–Meier analysis, unlike the analysis of Ghezalayagh et al., revealed that the presence of any TP53 mutation, and specifically an LOF mutation, was associated with a significantly lower PFI. Moreover, we found that a specific TP53 mutation, namely an LOF mutation, was predictive of PFI in our multivariate regression analysis. Therefore, our results, when compared to those of Ghezalayagh et al., suggest novel implications for patients with HGSC, warranting further investigations.

Consistent with the above clinical results, a preclinical in vitro study demonstrated a heightened efficacy of cisplatin in OC cells harboring mutant TP53 compared to those with wild-type TP53 [34].

Vice versa, a study by Kang et al. [31] found a greater likelihood of platinum treatment resistance and distant metastases in patients with HGSOC characterized by GOF TP53 mutations. Regarding the correlation of p53 mutations with survival outcomes, they showed a difference between recurrence patterns in OC with wild-type and GOF TP53 mutations but no change in overall or progression-free survival. Additionally, Zhang et al. [35], in a population of advanced HGSOC candidates for NACT or PDS, found that the TP53 K351N mutation was an independent factor for resistance to platinum-based chemotherapy and shorter DFS in patients who underwent NACT-IDS. Reles et al. found that TP53 loss of function due to inactivating mutations is shown to confer platinum resistance in ovarian tumor cells [36].

In only partial accordance with some of the above-cited studies, we found a notable disparity in platinum sensitivity between the functional categories of p53 mutations. However, unlike Kang et al. [31], we found that nonsense/frameshift/splicing mutations and LOF mutations were associated with a significantly lower percentage of platinum-sensitive patients compared with missense mutations and GOF mutations, respectively. Moreover, the LOF mutation of p53 was an independent negative predictor of platinum sensitivity in our multivariate regression analysis. LOF mutations were consistently associated with a significantly lower PFI compared to GOF mutations in our Kaplan–Meier analysis and multivariate COX regression analysis.

Among the BRCA-mutated HGSOC patients, no significant difference in platinum sensitivity or the PFI was observed between the different functional and structural p53 mutations. However, it is noteworthy that in five cases, LOF mutations of p53 were associated with null expression of HIC p53 in patients who exhibited refractoriness to platinum-based chemotherapy.

In this regard, Biatta et al. [14] shed light on how p53-null mutations are associated with a poorer prognosis and increased aggressiveness of HGSOC. Analyzing 34 cases of HGSOC, their study revealed that all cases with null immunohistochemical p53 expression exhibited TP53 mutations, which were linked to a significant reduction in overall survival (OS). HGSOCs characterized by the complete absence of p53 expression were associated with unfavorable outcomes. Similarly, Shahin et al. demonstrated that HGSOCs harboring TP53 null mutations had an elevated risk for tumor-related death [HR 2.17 (1.35–3.51)] compared to those with TP53 missense mutations [37]. However, these works, differently from our present one, did not consider stratification according to BRCA status and did not assess the association between TP53 mutation and response to platinum and the PFI. Therefore, the strength and novelty of our study was the finding of a significant association between a specific functional mutation, i.e., LOF, and an inferior PFI in the BRCA WT group. Of novelty, we found that the association between TP53 mutational status and a lower PFI was also maintained in BRCA mutant patients. Such results warrant confirmation in a larger cohort of BRCA-MT patients.

The disparate findings reported in the literature may be due to population or cohort heterogeneity but are also likely related to the different methods used to classify and describe TP53 mutations. TP53 mutations are classified using different schemes based on their frequency in cancer, functional activity, or structural protein changes [30]. There has been an increasing interest in mutations that confer oncomorphic or GOF activity on p53 rather than just nullifying the tumor suppressor function; however, there is no consensus on what defines a GOF mutation [31]. Differences in classification schemes may have led to conflicting conclusions regarding whether TP53 mutations are associated with outcomes in ovarian carcinoma. Moreover, the inclusion of different ethnicities may have influenced the rate and type of mutations; in this regard, our study included only Caucasian women.

Moreover, as shown by laboratory studies, chemoresistance and sensitivity depend not only on one mutated gene but also on the complex interplay between different signaling pathways, transcription factors, and enzymes and depend on the wild-type or mutated function of various genes. Therefore, a complete understanding of chemoresistance requires the analysis of multiple signaling pathways. In this regard, in ovarian cancer and in BRCA-mutated OC that are often sensitive to platinum-containing chemotherapy, further studies into the effect of TP53 must consider these interactions [38]. Our data are preliminary, considering the small number of patients and limited median follow-up period. Nevertheless, they suggest that HGSOC harboring p53 LOF and null mutations represents the poorest prognostic subgroup, especially regarding platinum sensitivity and the PFI. These patients may benefit from differentiated therapeutic protocols with close follow-up and more aggressive treatments. Noteworthy, alternative rationale therapeutic regimens may include immunotherapy with immune checkpoint inhibitors and mammalian target of rapamycin (mTOR) inhibitors. Indeed, preclinical evidence suggests that loss of p53 is associated with regulating immune response [39], PD-L1 expression, and activation of the mTOR pathway [40]. Moreover, inactivating mutations of p53 may lead to genomic instability [41], thus being potentially associated with increased efficacy of immunotherapy [42,43]. Moreover, several therapeutic approaches are under development for boosting p53 activity in cancer and targeting p53 pathway functional restoration in p53-mutated cancer [44].

Our results are encouraging and could serve as the foundation for a larger multicenter study to confirm our preliminary results in a larger sample size.

## 4. Materials and Methods

This study comprised a prospective cohort of 238 women aged ≥18 years diagnosed with primary ovarian, peritoneal, and fallopian tube carcinomas, collectively referred to as OC. These patients underwent surgery at the Department of Gynecologic Oncology, ARNAS G. Brotzu, Cagliari, Italy, between January 2019 and 31 December 2023. All participants provided informed consent for blood, surgical tissue, and clinical information collection, including long-term follow-up. Demographic and clinical characteristics, including primary histology, stage, grade, platinum response, and survival data at follow-up, were obtained by thoroughly reviewing the electronic patient record system. The International Federation of Gynecology and Obstetrics (FIGO) staging classification was used to determine the stage. Cancers were classified as platinum-sensitive or platinum-partially sensitive if the time to progression after adjuvant platinum therapy exceeded 12 months or 6 months, respectively. Cases not meeting these criteria were classified as platinum-resistant, including platinum-refractory. The study was approved by the Institutional Ethics Committee at the “ARNAS G. Brotzu” and “Azienda Ospedaliero Universitaria” in Cagliari (protocol code PG/2020/21663; date of approval 22 December 2020). Written informed consent for participation was obtained from all subjects involved in the study and for publication.

### 4.1. Somatic NGS Analysis

The NGS sequencing was performed on the genomic DNA extracted by paraffin-embedded tumor samples with at least 40% cancer cells. Noteworthy, the mean percentage of cancer cells was superimposable between the TP53 WT and TP53 MT samples (mean 41.8 versus 42.7). The DNA sequencing was performed with the NextSeq550 Illumina platform. Data were analyzed using the SOPHiA DDM Dx Homologous Recombination Deficiency Solution, Sophia Genetics, Lausanne, Switzerland. The analysis included the sequencing of 28 genes associated with homologous recombination repair (HRR), i.e., *AKT1*, *ATM*, *BARD1*, *BRCA1*, *BRCA2*, *BRIP1*, *CCNE1*, *CDK12*, *CHEK1*, *CHEK2*, *ESR1*, *FANCA*, *FANCD2*, *FANCL*, *FGFR1*, *FGFR2*, *FGFR3*, *MRE11*, *NBN*, *PALB2*, *PIK3CA*, *PPP2R2A*, *PTEN*, *RAD51B*, *RAD51C*, *RAD51D*, *RAD54L*, and *TP53*. The additional low-pass whole-genome sequencing (WGS) generated an index of genomic integrity ranging from −20 and +20, where a value <0 indicates a high genomic integrity and a value >0 indicates a low genomic integrity. The analysis also included the detection of the somatic SNP and INDEL as the amplification of BRCA1, BRCA2, and other genes involved in the HRR. Cases were classified as carrying a homologous recombination deficiency (HRD) mutation if testing revealed deleterious germline or somatic BRCA1 and BRCA2 mutations or mutations in other homologous recombination genes (*ATM*, *BARD1*, *BRIP1*, *NBN*, *PALB2*, *RAD51C*, and *RAD51D*).

Gene variants are described according to the International Standard Nomenclature of the Human Genome Variation Society and classified according to the international guidelines of the American College of Medical Genetics and Genomics [45]: “pathogenic”, “likely pathogenic”, and “of uncertain significance” are reported. Only the variant with a variant allele frequency (VAF) >5% was considered.

### 4.2. p53 Analysis and Classification

TP53 mutations were classified by structural and functional categories [38] to determine whether one classification was better associated with the outcomes (Table 6).

TP53 mutations were categorized as GO GOF or LOF mutations if there was enough evidence to support gain-of-function activity or loss-of-function activity, respectively, in human cell lines according to the International Agency for Research on Cancer TP53 database (http://p53.iarc.fr/ (accessed on January 9 2025), which is currently hosted by the ISB-CGC, a component of the NCI Cancer Research Data Commons (https://tp53.isb-cgc.org (accessed on January 9 2025). Utilizing three categories of GOF activity, which include (1) interference with p73 activity, (2) transactivation of genes repressed by wild-type p53, and (3) cooperation with oncogenes for the transformation of rat embryonic fibroblasts or mouse embryonic fibroblast cells, mutp53 was classified as having a GOF mutation if it met at least one of the three criteria [33].

### 4.3. Statistical Analysis

Comparative testing with chi-squared or Fisher’s exact tests examined TP53 mutation distribution and associations with clinicopathologic factors. Logistic regression analysis was used to determine the correlation between TP53 mutation and the platinum sensitivity and first platinum-free interval, defined as the time to radiologically confirmed relapse after the end of the first platinum-based treatment. A multivariate COX regression analysis was performed to assess the effects of TP53 mutation type on the PFI. Models were adjusted by stage and optimal cytoreduction. Kaplan–Meier and log-rank analyses compared the PFI based on TP53 mutation status. The BRCA mutation status was used to stratify the analysis and investigate the specific effects of the TP53 mutation. All analyses were performed using two-sided tests with a 5% type-I error rate. Statistical significance was set at a two-tailed *p*-value of < 0.05. All statistical analyses were performed using the MedCalc Statistical Software version 20.115 (2022 MedCalc Software Ltd., Ostend, Belgium).

## 5. Conclusions

A precise understanding of the inherent or acquired mechanisms of chemoresistance is crucial for optimizing the clinical management of HGSOC. Unraveling the various pathways involved in platinum resistance holds promise for developing alternative therapies that enhance existing platinum-based treatments by targeting these resistance pathways. Next-generation sequencing (NGS) enables the simultaneous assessment of multiple genomic and transcriptomic alterations. Therefore, identifying molecular biomarkers associated with platinum resistance could significantly improve the clinical management of HGSOC by facilitating the early prediction of inherent resistance to guide therapeutic selection. TP53 mutation could serve as a biomarker of inherent or acquired platinum resistance and aid in therapeutic planning. However, a more precise understanding of its function according to the specific type of mutation and in different molecular contexts is required before clinical exploitation. Only with a deeper knowledge of p53 biology will it be possible to develop targeted drugs against this critically important protein for developing HGSOCs.

## Figures and Tables

**Table 1:** Patients’ demographic and clinical characteristics according to BRCA mutational status.

	Total (No. 159)	BRCA1/BRCA2 Mutated (No. 44)	BRCA Wild Type (No. 115)	*p* Value
Age at diagnosis, years: mean ± SD	62.7 ± 12.3	61.5 ± 9.7	65.5 ± 10.8	0.0547
Stage, No. (%) II III IV	10 (6.3) 85 (53.4) 64 (40.2)	5 (11.4) 21 (47.7) 18 (40.9)	9 (10) 47 (50) 37 (40)	0.9324
Grade, No. (%) 3	159 (100)	44 (100)	93 (100)	NA
Primary optimal cytoreduction, No. (%) Suboptimal cytoreduction, No. (%)	118 (74) 41 (26)	33 (75) 11 (25)	85 (74) 30 (26)	0.8889
TP 53, No. (%) Wild Type Somatic mutation NA	22 (13.8) 131 (82.4) 6 (3.8)	4 (9.1) 38 (86.4) 2 (4.5)	18 (15.7) 93 (80.9) 4 (3.4)	0.5480
Other HRD genes Wild Type Somatic mutation NA	137 (86.1) 16 (10.1) 6 (3.8)	38 (86.4) 4 (9.1) 2 (4.5)	99 (86.1) 12 (10.4) 4 (3.5)	0.9261
Platinum response Sensitive Resistant/refractory	92 (57.9) 67 (42.1)	32 (72.7) 12 (27.3)	60 (52.2) 55 (47.8)	0.00192
PFI, months: mean ± SD (range)	12.2 ± 8.1 (1–41)	13.2 ± 6.3 (1–41)	11.7 ± 8.7 (1–24)	0.2986

Abbreviations: HRD, homologous recombination deficiency; PFI, platinum-free interval; SD, standard deviation; NA, not available. Significance was calculated with a chi-squared test and Student’s *t*-test. Results are significant for *p*-values less than 0.05.

**Table 2:** HRD mutations encountered in the cohort of patients.

Case	Gene	Mutation	VAF (%)
1	RAD51D (NM_002878.4)	c.479A>G, p.(Gln160Arg)	28.3
2	CCNE1 (NM_0001238)	c.1057G>A, p.(A353T)	35.4
3	BRIP1 (NM_032043.2)	c.656G>T, p.(Cys219Phe)	64
4	ATM (NM_000051.4)	c.3331C>G, p.(Leu1111Val)	31.2
5	PALB2 (NM_024675.4)	c.127A>G, p.(Lys43Glu)	28.6
6	BARD1 (NM_ 000465.3)	c.1052C>T, p.(Thr351Met)	43.3
7	CHEK2 (NM_007194.3)	c.320-5T>A, p. ?	59.3
8	CDK12	c.3446_3448dup, p.(Gln1149dup)	44.8
9	RAD54L	c.604C>T, p.(Arg202Cys)	38.6
10	RAD51D (NM_002878)	c.694C>T, p.(Arg232)	29.4
11	ATM (NM_000051)	c.2376+74_2476-314delinsTG, p. ?	30.3
12	CHEK2 (NM_007194)	c.1110del10, p.(Thr367fs)	42.1
13	CSEIL (NM_001362762.2)	c.936+1G>C, p. ?	26
14	RAD51D (NM_002878.4)	c.694C>T, p.(Arg232)	32
15	ATM (NM_000051)	c.8876_8879del, p.(Asp2959fs)	29.3
16	BRIP1 (NM_032043)	c.3262C>T, p.(His1088Tyr)	31.2

?: Indicates that the impact, nature, or consequences of this particular variant are not yet fully understood or remain uncertain.

**Table 3:** Comparison of PFI and platinum sensitivity distribution between different TP53 mutation types among BRCA WT and BRCA-MT patients.

	P53 Mutational Status			Structural Classification of P53 Mutation			Functional Classification of p53 Mutation			Hotspot Mutation		
Evaluated Patients	Wild Type (No. 22)	Mutated (No. 131)	*p* Value	Missense (*n* = 94)	Nonsense/ Frameshift/ Splice (*n* = 37)	*p* Value	GOF (*n* = 70)	LOF (*n* = 43)	*p* Value	Yes (*n* = 46)	No (*n* = 18)	*p* Value
All patients Platinum sensitive Platinum resistant/refractory PFI (months): mean ± SD	18 (81.8%) 4 (18.2%) 15.7 ± 6.4	70 (53.4%) 61 (46.6%) 9.6 ± 6.3	0.0130 <0.0001	57 (60.6%) 37 (39.4%) 9.5 ± 4.2	14 (37.8%) 23 (62.2%) 9.8 ± 4.3	0.0188 0.7153	40 (57.1%) 20 (28.6%) 12.7 ± 5.7	16 (37.2%) 27 (62.8%) 8.8 ± 4.2	0.0032 0.0002	27 (58.7%) 19 (41.3%) 8.3 ± 4.5	5 (27.8%) 13 (72.2%) 16.1 ± 6.3	0.0273 0.0541
Germline/somatic *BRCA* mutation Platinum sensitive Platinum resistant/refractory PFI (months): mean ± SD	4 (18.2%) 4 (18.2%) 0 (0%) 19 ± 4.3	38 (29%) 28 (21.4%) 10 (7.6%) 12 ± 6.1	0.2940 * 0.2455 0.0318	29.8 (29.8%) 22 (23.4%) 6 (6.4%) 10.8 ± 6	10 (27%) 6 (16.2%) 4 (10.8%) 14.1 ± 4.5	0.7746 * 0.2586 0.1595	19 (27.1%) 15 (21.4%) 4 (5.7%) 12.8 ± 5.2	10 (23.3%) 6 (14%) 4 (9.3%) 12.8 ± 5.2	0.6475 * 0.2863 0.1121	20 (43.5%) 14 (30.4%) 6 (13%) 12.1 ± 6.4	3 (16.7%) 2 (11.1%) 1 (5.5%) 12.1 ± 6.4	0.0461 * 0.9089 0.3186
Cases without *BRCA* mutation Platinum sensitive Platinum resistant/refractory PFI (months): mean ± SD	18 (81.8%) 14 (63.6%) 4 (18.2%) 14.3 ±6.4	93 (71%) 42 (32.1%) 51 (38.9%) 8.3 ± 3.1	0.0117 <0.0001	66 (70.2%) 35 (37.2%) 31 (33%) 8.5 ± 3.4	27 (73%) 7 (18.9%) 20 (51.4%) 8.1 ± 3.5	0.0177 0.6364	51 (72.9%) 30 (42.9%) 21 (30%) 12.3 ± 5.9	33 (76.7%) 10 (23.3%) 23 (53.4%) 7.9 ± 2.1	0.0111 0.0001	26 (56.5%) 13 (28.3%) 13 (28.3%) 8.8 ± 4.7	15 (83.3%) 3 (16.7%) 12 (66.7%) 7.8 ± 1.3	0.0610 0.3793

Abbreviations: WT, wild type; MT, mutant; PFI, platinum-free interval. Significance was calculated with Fisher’s exact test and Kruskal–Wallis test. Results are significant for *p*-values less than 0.05; * *p*-value calculated versus cases without BRCA mutations.

**Table 4:** Logistic regression analysis examining effects of *TP53* mutation type on platinum sensitivity based on *TP53* classification scheme and according to BRCA mutation status.

	Regression Coefficient	Hazard Ratio	95% Confidence Interval	Wald	*p* Value
TP53 mutational status Wild type Mutated BRCA WT BRCA MT	Reference −2.23232 −2.31785 −1.09861	0.1073 0.0985 0.3333	0.0127–0.9030 0.0106–0.9116 0.0310–3.5789	4.2184 4.1678 0.8229	0.0400 0.0412 0.3643
Structural classification					
Wild type	Reference				
Missense BRCA WT BRCA MT	−0.76564 −0.98083 −0.87547	0.4650 0.3750 0.4167	0.1540–1.4041 0.1005–1.3993 0.0373–4.6568	1.8442 2.1313 0.5053	0.1745 0.1443 0.4772
Nonsense/frameshift/splice BRCA WT BRCA MT	−1.72050 −2.15948 −1.28093	0.1790 0.1154 0.4167	0.0550–0.5820 0.1005–1.3993 0.0216–3.5772	8.1788 8.3860 0.9652	0.0042 0.0038 0.3259
Functional classification					
Wild type	Reference				
Loss of function BRCA WT BRCA MT	−1.60776 −1.82813 −1.50408	0.2003 0.1607 0.2222	0.0705–0.5695 0.0474–0.5448 0.0208–2.3699	9.0962 8.6158 1.5513	0.0026 0.0033 0.2130
Gain of function BRCA WT BRCA MT	0.55962 0.40547 0.15415	1.7500 1.5000 1.1667	0.3102–9.8727 0.1383–16,2686 0.0593–22.9378	0.4019 0.1111 0.0103	0.5261 0.7388 0.9192
Hotspot classification					
Wild type	Reference				
Hotspot missense BRCA WT BRCA MT	−0.64870 −0.82668 −1.01857	0.5227 0.4375 0.3611	0.1865–1.4649 0.1256–1.5238 0.0352–3.7025	1.5223 1.6859 0.7357	0.2173 0.1941 0.3911
Other mutation BRCA WT BRCA MT	−1.88707 −2.12596 −1.09861	0.5227 0.1193 0.3333	0.0405–0.5674 0.0252–0.5651 0.0136–8.1830	7.8473 7.1787 0.4526	0.0051 0.0074 0.5011

Abbreviations: WT, wild type; MT, mutated. Results are significant for *p*-values less than 0.05.

**Table 5:** Multivariate COX regression analysis of the variables associated with the PFI.

	Adjusted HR	95% CI	*p*-Value
Univariate			
TP53 mutation (yes/no)	5.4242	0.7321–40.1908	0.0980
TP53 structural classification (yes/no) missense nonsense/frameshift/splicing	2.4978 3.0828	0.9206–6.7772 1.1651–8.1565	0.0723 0.0233
Functional classification (yes/no) LOF GOF	3.3455 0.9904	1.3484–8.3002 0.1996–4.9144	0.0092 0.9906
Hotspot classification (yes/no) hotspot missense other mutations	2.0941 2.9940	0.8428–5.2027 1.1076–8.0928	0.1114 0.0307
Multivariate (stepwise method)			
Functional classification LOF	3.3537	1.4857–7.5700	0.0036

Abbreviations: LOF, loss of function; GOF, gain of function. Models were adjusted by stage and optimal cytoreduction.

**Table 6:** Mutation classification categories.

	Structural Classification	Functional Classification
Definition	Standard definitions of mutation types based on data from the UMD TP53 database https://p53.fr/tp53-database (accessed on January 9 2025)]	Nonsense/frameshift/splice mutations were considered LOF mutations. Missense mutations were further subdivided into LOF or GOF based on ref [33]
Categories	Wildtype Missense Nonsense/frameshift/splice	Wild type Hotspot missense Other mutations

Abbreviations: LOF, loss of function; GOF, gain of function.

## Data Availability

Original clinical, laboratory, and instrumental data can be found in the patient charts archived at the Clinical Department and the Data Management Service at the Department of Medical Oncology at the University of Cagliari. The data generated in the present study may be requested from the corresponding author.

## Supplementary Materials

The supporting information can be downloaded at https://www.mdpi.com/article/10.3390/ijms26052232/s1.

## Abbreviations

HGS-OC	high-grade serous ovarian cancer
PS	platinum sensitivity
PFI	platinum-free interval
MT	mutated
WT	wild-type
LOF	loss of function
GOF	gain of function
PARPi	PARP inhibitor
PFS	progression-free survival
OS	overall survival
HR	homologous recombination
NGS	next-generation sequencing
HRR	homologous recombinant repair
HRD	homologous recombination deficiency
