# Supplementary material for: Association Between TP53 Mutations and Platinum Resistance in a Cohort of High-Grade Serous Ovarian Cancer Patients: Novel Implications for Personalized Therapeutics
Source: Int J Mol Sci. 2025 Mar 1;26(5):2232. doi: 10.3390/ijms26052232 (PMC11901047; doi:10.3390/ijms26052232)
Supplement: Supplementary file 1 [file ijms-26-02232-s001.zip › ijms-3446498-supplementary.pdf]

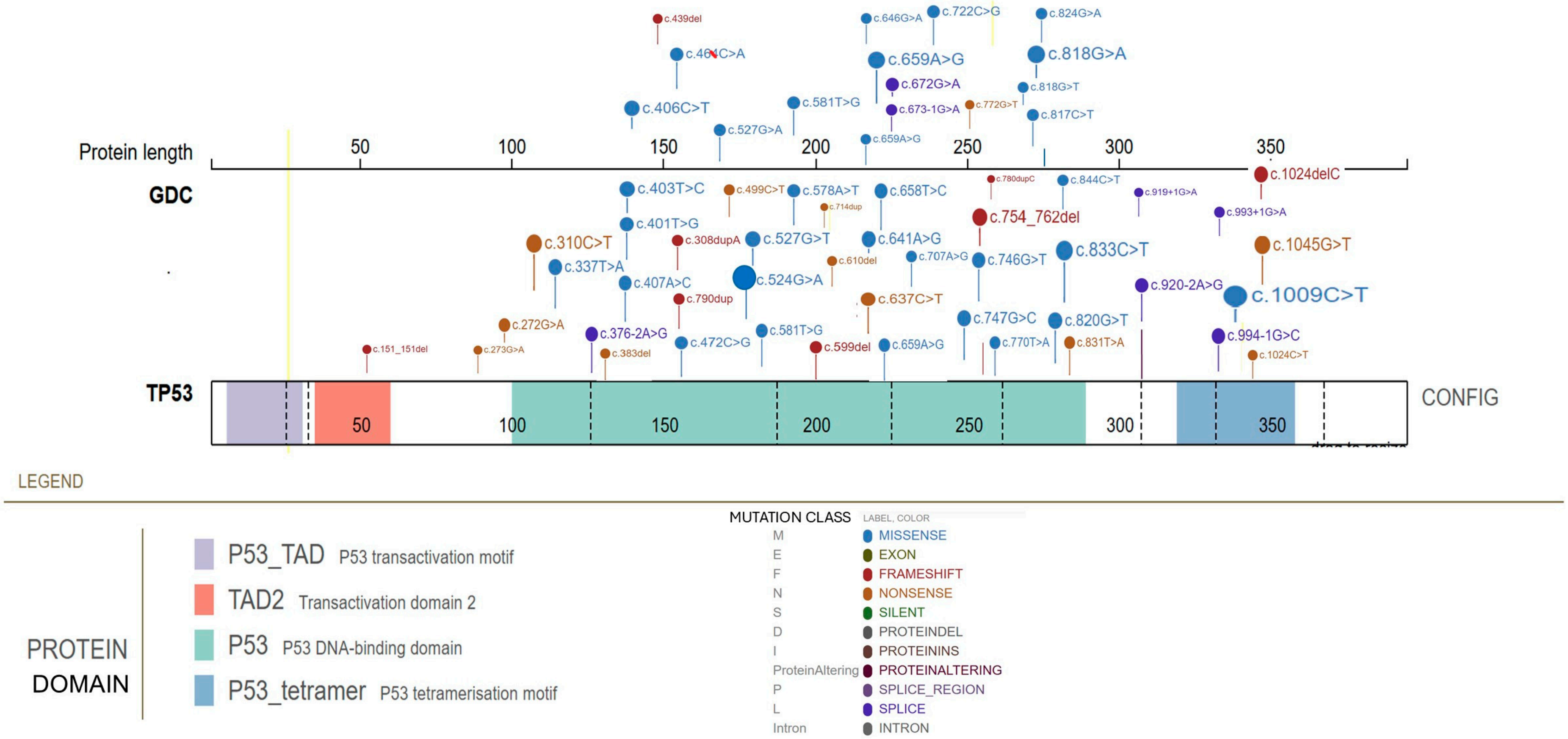

Figure S1.Lollipop plot showing the location and frequency of p53 mutations in our cohort

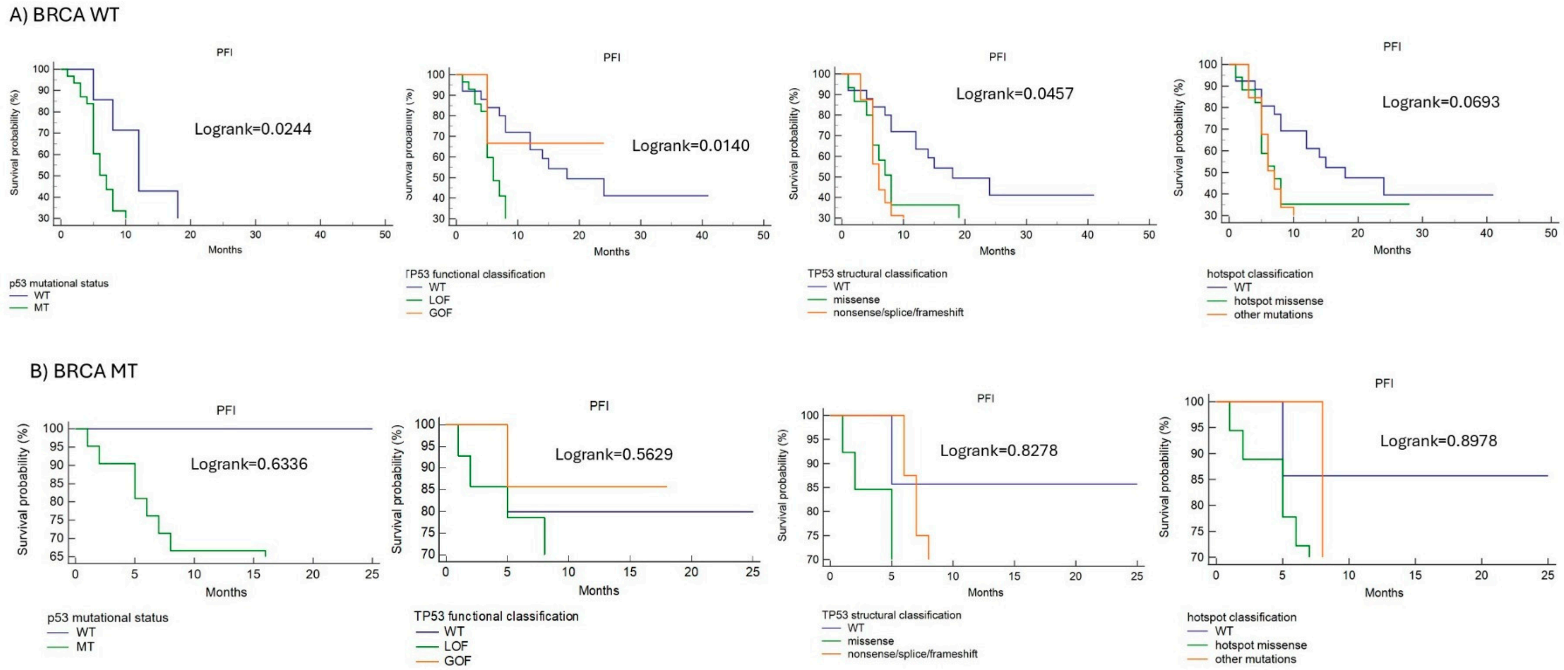

Figure S2: Platinum-free interval comparison according to TP53 mutational status among patients stratified by the presence of germline or somatic BRCA mutations
